# 

**Published:** 1844-05

**Authors:** 


### CONTENTS

OF NO. V.

### ORIGINAL COMMUNICATIONS.

### ESSAYS AND CASES.

Art. I.—A case of Hepatic Abscess, which discharged pus at three points. By Andrew R. Kilpatrick, M. D., of Woodville, Miss. - - - - - - - - - 377

Art. II.—A case of Tubercular Disease. By W. W. Goodwin, M.D., of Utica, Indiana. - - - - - - - - 382

Art. III.—A case of Stricture of the Urethra, with effusion of urine into the Scrotum, sloughing and loss of a Testis; and a case of Epilepsy in which trephining was resorted to. By Wm. M. Yandell, M.D., of Benton, Miss. - 384

Art. IV.—Typhus Abdominalis; Ganglianic Typhus; Nervous Fever. From Prof. Schoenlein's Lectures, published by some of his disciples. - - - - - - - 386

Art. V.—Cancer of the inferior lip and corresponding maxillary bone; cure after two operations, performed at different periods. By Martial Dupierres, M.D., of Havana. 405

### REVIEWS.

Art. VI.—The Anatomy, Physiology and Pathology of the Human Teeth, with the most approved methods of Treatment; including Operations and the method of making and setting artificial teeth: with 30 plates. By Paul B. Goddard, M.D., M.A.N.S., M.A.P.S., Demonstrator of Anatomy in the University of Pennsylvania, Lecturer on Anatomy, &c., &c. Aided in the Practical part by Joseph E. Parker, Dentist. - - - - - - 410

Art. VII.—First Principles of Medicine. By Archibald Billing, M.D., A.M., Member of the Senate of the University of London, Fellow of the Royal College of Physicians, &c., &c. First American from the Fourth London Edition. - - - - - - - - 421
ART. VIII.—De Voce, Lingua, Respiratione, Deglutitione Observationes quædam. Dissertatio Inauguralis Physiologica quam ex auctoritate et consensu gratiosi medicorum ordinis in alma literraum Universitate Fredericia Guilielmia Rhenana ad Doctoris Medicine et Chirurgiæ, gradum rite assequendum scripsit et die V. Aprilis anni MDCCCXXXXI publice defendit CAROLUS ERNESTUS NOEGGERATH, Rhenano-Borussus. Cum tabula lithographica. Bonnæ, typis Caroli Georgii, MDCCCXLI. - - - - - 431
ART. IX.—A Treatise on Food and Diet, with Observations on the Dietetical Regimen suited for the Disordered state of the Digestive Organs, &c. By JONATHAN PERIERA, M.D., F.R.S., and L.S., &c., &c. Edited by CHARLES A. LEE, M.D. - - - - - - - - 440

BIBLIOGRAPHICAL NOTICES.

ART. X.—The Anatomy and Surgical Treatment of Abdominal Hernia: with numerous plates. By SIR ASTLEY COOPER, Bart., F.R.S., Surgeon to the King, and Consulting Surgeon to Guy's Hospital. From the Second London Edition. By C. ASTON KEY, Senior Surgeon to Guy's Hospital and Lecturer on Surgery. - - - - - . 448
ART. XI.—Human Physiology. With upwards of three hundred illustrations. By ROBLEY DUNGLISON, M. D., Professor of the Institutes of Medicine, etc., in Jefferson Medical College, Philadelphia, &c., &c. Fifth Edition, greatly modified and improved. - - - - - - 450

SELECTIONS FROM AMERICAN AND FOREIGN JOURNALS.

Medicines Administered by the Nose. - - - - - - 451
Judicial Advice to Medical Practitioners. - - - - 453
Statistics of Midwifery. - - - - - - - 454
On Tubercles and Phthisis. - - - - - - : 455

ORIGINAL INTELLIGENCE.

Philadelphia Hospitals. - - - - - - - 459
Transylvania Medical School. - - - - - - 460
Dr. Patterson's Valedictory Address. - - - - - - 463
Wounds of the Intestines treated by Suture—recovery. - 465
Lunatic Asylum in Indiana. - - - - - - - 466
Medical Lectures. - - - - - - - - - 467
London Lancet. - - - - - - - - - 467
Books and Communications Received. - - - - - 468

